# Immobilization of Enzymes on a Phospholipid Bionically Modified Polysulfone Gradient-Pore Membrane for the Enhanced Performance of Enzymatic Membrane Bioreactors

**DOI:** 10.3390/molecules23010144

**Published:** 2018-01-11

**Authors:** Yizong Guo, Xueyan Zhu, Fei Fang, Xiao Hong, Huimin Wu, Dajing Chen, Xiaojun Huang

**Affiliations:** 1 MOE Key Laboratory of Macromolecular Synthesis and Functionalization, Department of Polymer Science and Engineering, Zhejiang University, Hangzhou 310027, China; yzguo@zju.edu.cn (Y.G.); 21629001@zju.edu.cn (X.Z.); 3090102659@zju.edu.cn (F.F.); 11629013@zju.edu.cn (X.H.); wuhuimin1224@163.com (H.W.); 2 College of Biomedical Engineering & Instrument Science, Zhejiang University, Hangzhou 310027, China

**Keywords:** enzymes, phospholipids, biomimetic interfaces, gradient-pore membrane, membrane bioreactors

## Abstract

Enzymatic membrane bioreactors (EMBRs), with synergistic catalysis-separation performance, have increasingly been used for practical applications. Generally, the membrane properties, particularly the pore structures and interface interactions, have a significant impact on the catalytic efficiency of the EMBR. Therefore, a biomimetic interface based on a phospholipid assembled onto a polysulfone hollow-fiber membrane with perfect radial gradient pores (RGM-PSF) has been prepared in this work to construct a highly efficient and stable EMBR. On account of the special pore structure of the RGM-PSF with the apertures decreasing gradually from the inner side to the outer side, the enzyme molecules could be evenly distributed on the three-dimensional skeleton of the membrane. In addition, the supported phospholipid layer in the membrane, prepared by physical adsorption, was used for the immobilization of the enzymes, which provides sufficient linkage to prevent the enzymes from leaching but also accommodates as many enzyme molecules as possible to retain high bioactivity. The properties of the EMBR were studied by using lipase from *Candida rugosa* for the hydrolysis of glycerol triacetate as a model. Energy-dispersive X-ray and circular dichroism spectroscopy were employed to observe the effect of lecithin on the membrane and structure changes in the enzyme, respectively. The operational conditions were investigated to optimize the performance of the EMBR by testing substrate concentrations from 0.05 to 0.25 M, membrane fluxes from 25.5 to 350.0 L·m^−2^·h^−1^, and temperatures from 15 to 55 °C. As a result, the obtained EMBR showed a desirable performance with 42% improved enzymatic activity and 78% improved catalytic efficiency relative to the unmodified membrane.

## 1. Introduction

Enzymatic membrane bioreactors (EMBRs), combined with enzyme immobilization and membrane technology, exhibit synergistic catalysis and separation by integrating the high catalytic efficiency of enzymes with the high selectivity of membrane separation; this technique has shown great potential in many fields, such as the food industry, chemical manufacturing, and environmental purposes [1,2,3,4,5,6]. Nevertheless, there are still some concerns and urgent problems to be solved in the future research and application of EMBRs, one of the most crucial of which is the enhancement of the catalytic properties [7,8,9]. The performance of EMBRs is mainly dependent on the enzyme loading and activity, which are intricately connected with the microstructure and microenvironment of the supporting membrane, respectively [10,11,12]. It is well known that high enzyme loading can greatly promote the catalytic efficiency of EMBRs and thus, many relevant methods have been developed in the past decades [9,13]. Some researchers focused on the immobilization behavior of the enzyme, including chemical bonding, physical adsorption, and entrapment [13,14,15]. Others studied ways to immobilize as many enzyme molecules as possible through surface modification of the membrane [9,16]. However, the decrease in enzyme activity induced by aggregation of the enzyme molecules is often ignored [12,17]. On the other hand, the immobilization of enzymes on the membrane is a process in which the enzyme molecules will inevitably interact with the membrane surface, which leads to a conformation change in the enzyme and its partial inactivation, as has been proved by many reports that the activity of immobilized enzymes tends to be lower than that of free enzymes [18,19,20]. Therefore, it is of great practical significance to construct a biologically friendly microenvironment to reduce the interaction between the enzyme and the supporting membrane to prevent enzyme inactivation.

The important properties during the choice of a membrane are appropriate pore structure and permeability, which are vital to enhance the amount of enzyme loading and the timely separation of the product [21,22,23]. Therefore, membranes with perfect radial gradient pores, the size of which reduces gradually from the inner side to the outer side, meet the demands and hence, have been selected as supports. Through simple dead-end filtration, enzyme molecules can be immobilized equably in the membrane pores by hydrophobic interactions, thereby improving the enzyme loading and stability relative to immobilization on the membrane surface [24]. Moreover, some researchers have tried to fix enzymes in membrane pores with homogeneous structures in order to increase the amount of enzyme loading, although the accompanying problem that the enzyme molecules are inclined to block the pores and cause membrane fouling needs to be solved [25,26]. By contrast, polysulfone (PSF) membranes with radial gradient pore structure (RGM-PSF) have advantages that greatly increase the number of enzyme immobilization sites and allow the fixation of enzyme molecules in the three-dimensional network structure of the membrane; this effectively reduced the aggregation of the enzyme and gave excellent performance, including high flux, low osmosis resistance, and outstanding catalytic efficiency [27].

In addition, there is the challenge that the activity of enzymes tends to decrease after the immobilization process because of the variation of the enzyme configuration caused by interactions between the enzyme molecules and the support [28]. It is therefore of great importance to reduce the abiotic interface interaction between them. One of the most promising routes to solve this problem is to immobilize enzymes in as similar a manner as possible to those found in biological systems [12]. The concept of biomimetism is based on inspiration from the fluid mosaic model of the cell and tries to replicate this for use in enzyme immobilization. An interesting approach in this report involves molecular modification of membranes with phospholipid biological interfaces, which is expected to create ideal host matrices for the maintenance and enhancement of enzymatic activity.

In this paper, a facile method integrating a biomimetic interface and immobilization was developed, based on the cell fluid mosaic model. Briefly, a PSF hollow-fiber membrane with perfect radial gradient pores and low mass resistance was prepared as the support material. After modification with lecithin, which is one of the varieties of phospholipid, *Candida rugosa* lipase was immobilized on the membrane by simple dead-end filtration to prepare the EMBR. Meanwhile, energy-dispersive X-ray (EDX) and circular dichroism (CD) spectroscopy were employed to observe the effect of lecithin on the membrane and the structure changes of the enzyme molecules, respectively. In addition, to further study the performance of the prepared EMBR, hydrolysis of glycerol triacetate was used as a model, and various operational variables, such as membrane flux, substrate concentration, and temperature, were investigated to obtain the optimal experimental conditions.

## 2. Results and Discussion

### 2.1. Mimetic Biological Microenvironment Based on Enzyme Surfactant Micelles

**Figure 1:**
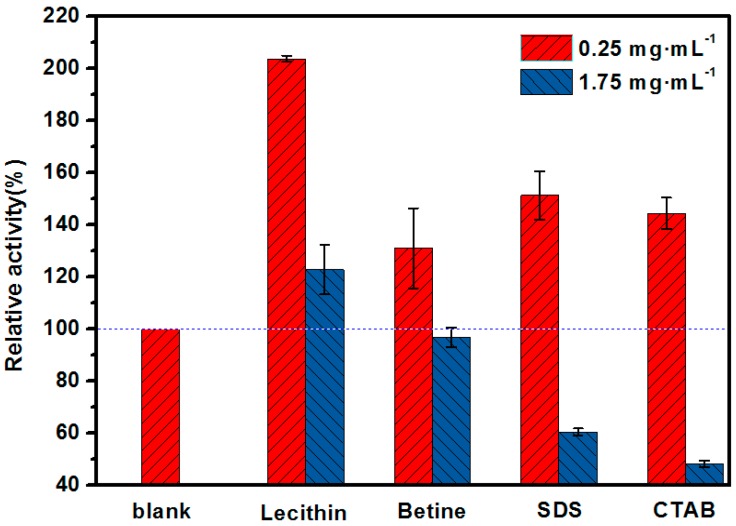
The effect of different charged surfactants on the activity of free lipase.

The construction of an in vitro biofriendly microenvironment plays an important role in maintaining the high catalytic activity of the enzyme, and surfactants are often used to form enzyme surfactant micelles that show significantly higher efficiency [29,30]. It has been confirmed that the higher activity is a result of the structure of the surfactant and the interaction between the micelle-bound enzyme and the substrate. To investigate the effects of different surfactants on the catalytic performance, four different surfactants were selected. The free lipase solution was doped with surfactant to form an enzyme surfactant complex before addition to the substrate solution. As shown in Figure 1, different surfactants had a significant influence on the catalytic activity of the lipase. At a low concentration of 0.25 mg·mL^−1^, all of the surfactants, whether zwitterionic (lecithin, betine), anionic (sodium dodecyl sulfate, SDS), or cationic (hexadecyltrimethyl ammonium bromide, CTAB), had a positive effect on the activity of the free lipase; among them, lecithin performed best and improved the activity of the free lipase to 203% relative to blank lipase solution. However, with the surfactants at a higher concentration of 1.75 mg·mL^−1^, the surfactants, except for lecithin, clearly deactivated the enzyme, and the relative activities were 96%, 59% and 45%, respectively.

This could be explained as follows: unlike charge balanced zwitterionic surfactants, surfactants that are solely positively or negatively charged have stronger electrostatic interactions with the enzyme molecules, which can change the optimum enzyme conformation and can even cause severe enzyme inactivation [29]. Furthermore, lecithin, as a natural part of the composition of cytomembranes, possesses good biocompatibility, which could provide a biologically friendly environment for the enzyme and reduce the non-biologically specific interactions between the enzyme and support. Therefore, lecithin was selected as the medium to construct a biomimetic biocompatible interface.

### 2.2. The Optimization of the Structure of the Enzyme Lecithin Complex

**Figure 2:**
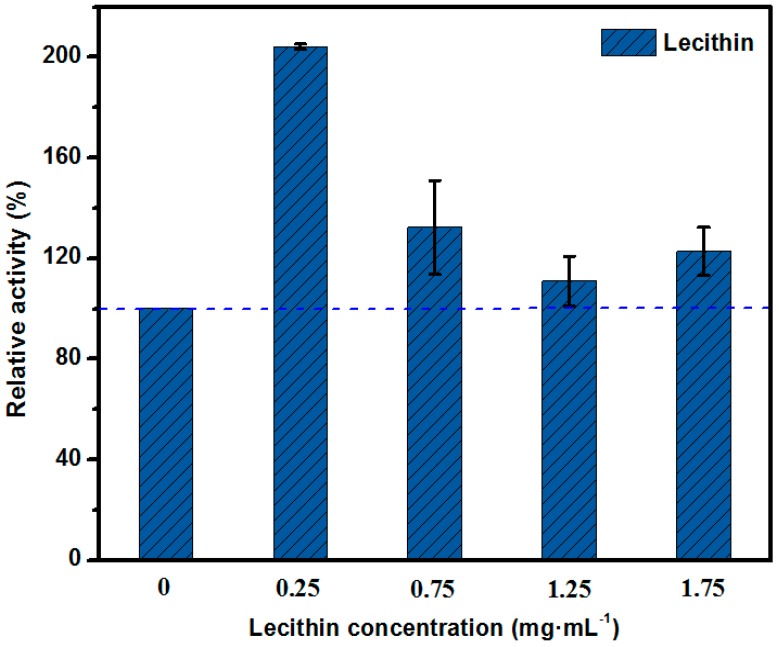
The effect of the concentration of lecithin on the relative activity of free lipase.

As indicated in Figure 1, the concentration of the surfactant has an important impact on the enzyme activity, and it is thus necessary to investigate the optimal concentration of lecithin. Figure 2 shows that the addition of lecithin at 0.25 mg·mL^−1^ doubled the relative activity of the free lipase, whereas a further increase in the concentration of lecithin dramatically decreased the relative activity, although the relative activity remained above 100%. This result can be explained by “induced fit” theory, which indicates that, before the enzyme binds to the substrate, the structure of the active center of the enzyme is not very fit for the substrate structure. After the enzyme is hit by the substrate, the enzyme conformation is induced to change slightly on account of the flexible and transformable active site. Thus, the active center and the substrate can form a perfectly complementary shape for the enzyme substrate complex, which causes the catalytic reaction [18,31]. The main type of interaction between the enzyme and lecithin is electrostatic, because of the large number of positive and negative charges on the surface of the enzyme and lecithin, which can alter the conformation and activity of the enzyme to some extent. In particular, at low concentrations, lecithin, contains a monovalent quaternary ammonium cation, binds to the lipase through weak electrostatic interactions, which alter the conformation of the enzyme and allow it to combine with the substrate more easily [30]. Nevertheless, as the concentration of lecithin increased, a decay phenomenon emerged in the relative activity of the lipase. This may due to the saturated adsorption between the hydrophobic segments of the lecithin and lipase, together with the negative effect of increased mass transfer resistance.

**Figure 3:**
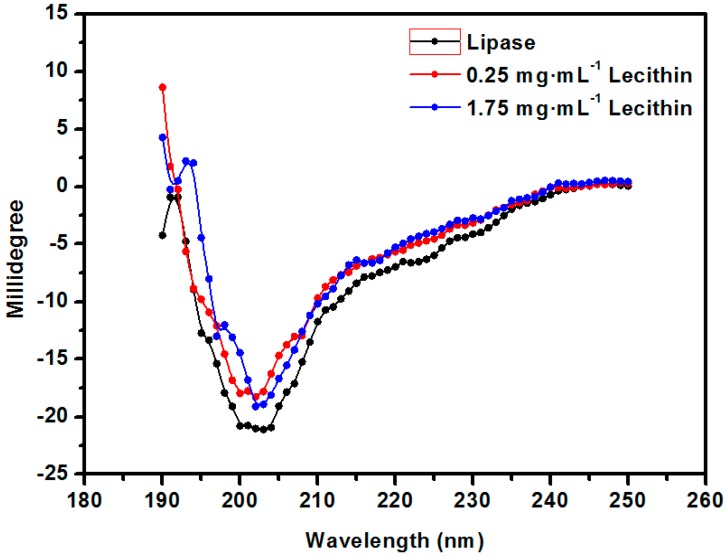
CD spectra of free lipase and lecithin-lipase complexation.

To further probe the effect of lecithin on the structure of the lipase, CD spectra were measured as indicated in Figure 3. A concentration of 0.25 mg·mL^−1^ lecithin had minimal effect on the structure of the lipase, whereas the shape of the adsorption peak changed to a large extent at 1.75 mg·mL^−1^, which was in agreement with the results in Figure 2. There are large amounts of hydrogen bonding in the α-helix, and β-sheet in the secondary structure of the protein, which make the protein rigid to some degree. Therefore, excessive α-helix structure hinders the exposure of the active center and decreases the activity of the enzyme. The α-helix structure of the protein is indicated by a positive peak at around 192 nm; no such peak appeared at a concentration of 0.25 mg·mL^−1^, whereas the peak was red-shifted and the intensity increased at a concentration of 1.75 mg·mL^−1^, which led to the decrease in lipase activity at the higher concentration of lecithin.

### 2.3. Characterization of the PSF Membrane with a Lecithin Biomimetic Interface

**Figure 4:**
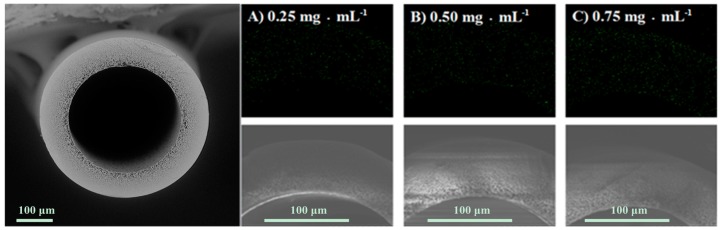
SEM image of blank PSF membrane (left) and the nitrogen distribution (measured by EDX) of the lecithin-modified PSF membrane with different concentrations of lecithin (**A**–**C**).

The morphology and properties of membrane might be changed after the modification with lecithin, which might affect the performance of the EMBR. Figure 4 shows that nitrogen was uniformly distributed in the skeleton of the lecithin-modified PSF hollow-fiber membrane. As indicated in Table 1, the adsorption capacity increased gradually with the increase in lecithin concentration. Meanwhile, no obvious changes were observed in the morphology of the membrane after modification with lecithin, although the average pore size decreased from 0.236 to 0.212 μm (Supplementary Materials Figure S1). The cross-section of the membrane presented perfect radial gradient pores, the size of which became smaller from the inner to the outer surface.

**Figure 5:**
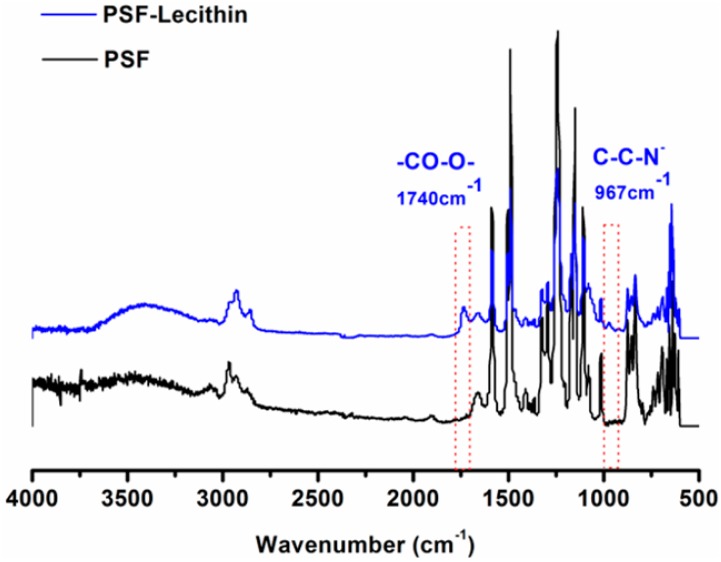
ATR-FTIR spectra of the blank and lecithin-modified RGM-PSF.

To further verify the adsorption of lecithin on the membrane, attenuated total reflectance Fourier transform infrared (ATR-FTIR) spectroscopy was implemented, with the results shown in Figure 5. In comparison with the spectrum of blank RGM-PSF, there were functional group peaks of C-C-N^−^ at 967 cm^−1^ and a stretching vibration absorption peak of C=O at 1740 cm^−1^, which provided evidence that lecithin was effectively absorbed onto the membrane by dead-end filtration.

### 2.4. The Effect of the Lecithin Biomimetic Interface on the Performance of the EMBR

**Figure 6:**
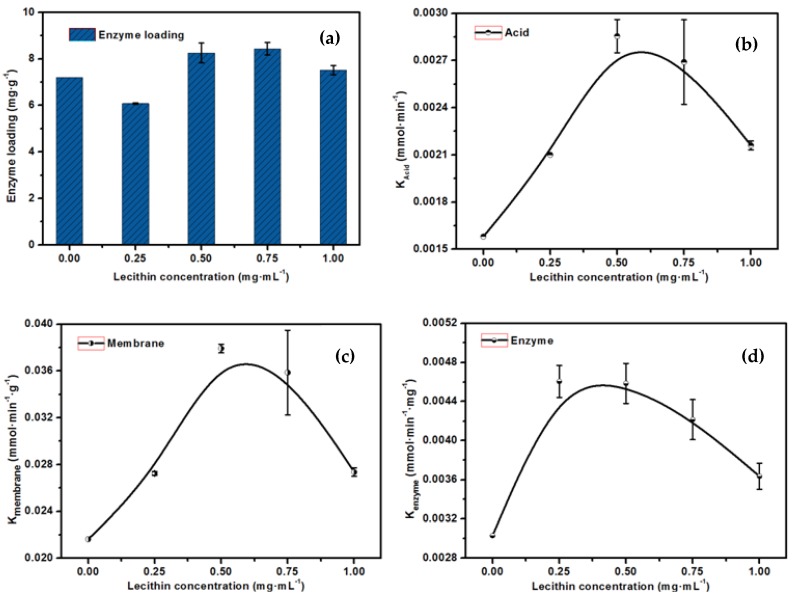
The effect of the concentration of the lecithin on the performance of the EMBR (**a**–**d**).

Figure 6a shows the amount of enzyme adsorbed with different lecithin concentrations, which indicates that, as the concentration of lecithin increased from 0.25 mg·mL^−1^ to 0.75 mg·mL^−1^, the enzyme loading increased from 6.1 mg·g^−1^ to 8.3 mg·g^−1^. The reason is as follows. Without lecithin, lipase was absorbed onto membrane through hydrophobic effects. After addition of a small amount of lecithin (0.25 mg·mL^−1^), the hydrophobic sites of lecithin occupied the adsorption sites on the membrane and its hydrophilic sites were exposed externally, which developed a single molecular layer of lecithin that interacted with the lipase by weak electrostatic repulsion and hydrophilic effects and led to a low enzyme loading. With the increase in the concentration of lecithin, a double molecular layer of lecithin formed, which was similar to the phospholipid bilayer of a cell, and lipase bound with the hydrophobic sites of the lecithin through hydrophobic effect, which resulted in an increased enzyme loading.

Abiotic specific interactions between the enzyme and the support often lead to inactivation of immobilized enzymes, which greatly affects the catalytic efficiency. Using glycerol triacetate as substrate, the hydrolysis reaction catalyzed by lipase occurred in the pores of the immobilized-lipase membrane, which produced quantities of acetic acid; the acid dispersed into the substrate solution and lowered the pH value. Figure 6b–d shows that the enzymatic lecithin-modified membrane had an optimal membrane activity of 0.0382 mmol·min^−1^·g^−1^ and enzyme activity of 0.0046 mmol·min^−1^·mg^−1^ at 0.5 mg·mL^−1^ lecithin; these values were much higher than the performance of the nascent membrane under the same experimental conditions and indicate the better biocompatibility of the lecithin-modified membrane. This result is similar to the result of the promoting role that the lecithin played under solution conditions (Figure 3). A low concentration of lecithin could adjust the flexibility of the lipase and promote its interaction with the substrate. However, excessive lecithin aggrandized the mass transfer resistance in the reaction process and caused a decrease in the activity of the EMBR.

### 2.5. The Effect of Ion Strength on the Performance of the EMBR

**Figure 7:**
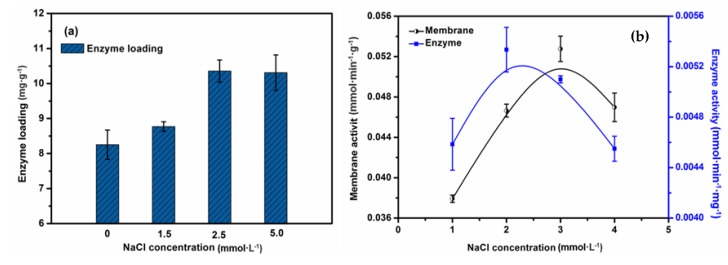
Effect of ion strength on the enzyme loading (**a**) and the activity of EMBR and enzyme (**b**).

The ion strength in the solution has a vital impact on the structure of enzymes and affects their catalytic activity. In PBS (pH 7.0), lecithin (isoelectric point, Ip 6.8) is slightly electronegative and lipase (Ip 4.6) carries a large amount of negative charge, which results in electrostatic repulsion between them and a decrease in the enzyme loading. Under such circumstances, we tried to introduce an electrolyte into the system to weaken the electrostatic repulsion because of the electrostatic shielding effect caused by electrolyte ions that neutralize the adsorbed sites with counter ions. As illustrated in Figure 7a, with an increase in the concentration of NaCl, the enzyme loading on the membrane increased and was raised by 25% under the optimum conditions. When the concentration of NaCl was higher than 2.5 mmol·L^−1^, the enzyme loading was saturated and did not increase further.

Meanwhile, it has been acknowledged that, apart from a relatively stable pH environment, a certain degree of ion strength is vital for high enzyme activity [31]. Figure 7b shows that the activity of the immobilized enzyme and EMBR increased first and then decreased with an increase in the concentration of NaCl; this is because extortionate ion strength may alter the secondary structure of the enzyme and even inactivate it. With regard to the observation that the optimum catalytic activity of EMBR was lagging behind that of the enzyme, it may that the high enzyme loading compensated for the effect of partial inactivation of the enzyme.

### 2.6. The Effect of the Concentration of Lipase on Enzyme Loading and Activity of the EMBR

**Figure 8:**
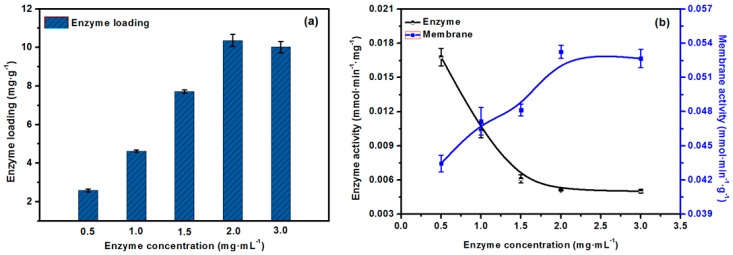
(**a**) Effect of initial concentration of lipase on the enzyme loading; (**b**) Effect of initial concentration of lipase on the activity of enzyme and EMBR.

To some extent, a high enzyme loading is conducive to enhanced catalytic efficiency of the EMBR. Figure 8a shows that, with an increase in the enzyme concentration, the enzyme loading increased gradually and reached maximum value of 10.7 mg·g^−1^ with a 2.0 mg·mL^−1^ lipase solution. Figure 8b shows that the activity of the lipase continually decreased from 0.017 to 0.0052 mmol·min^−1^·g^−1^ as the concentration of lipase increased from 0.5 mg·mL^−1^ to 3.0 mg·mL^−1^, whereas the activity of the EMBR increased from 0.043 to 0.053 mmol·min^−1^·g^−1^. On the one hand, the increase in enzyme concentration led to an increase in the amount of enzyme loaded on the membrane, which resulted in an accumulation of enzyme molecules in the membrane pores with limited adsorption sites; this greatly increased the mass-transfer resistance and reduced the conformation transition of the enzyme, thereby lowering the activity of the enzyme. On the other hand, with respect to the decrease in the activity of the enzyme, an increase in enzyme loading affected the whole EMBR more conspicuously. It is clear that, to a certain extent, a higher amount of enzyme loading can significantly increase the catalytic efficiency of the EMBR, which is beneficial for application of the EMBR at a large scale.

### 2.7. Optimization of the Properties of the EMBR

In addition to the simple preparation method of the EMBR described above, the catalytic conditions can be optimized in order to construct a highly efficient and controlled EMBR. Herein, the effect of membrane flux, substrate concentration, and temperature were investigated by the controlling-variables method.

**Figure 9:**
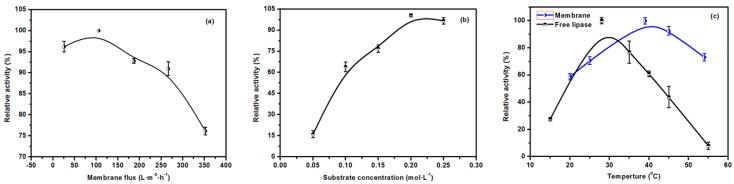
Effect of membrane flux (**a**); substrate concentration (**b**); and temperature (**c**) on the activity of the free and immobilized lipase.

As indicated in Figure 9a, at 25.5 L·m^−2^·h^−1^ of membrane flux, although the EMBR could react adequately with the substrate, the produced acid could not be quickly separated, which reduced the efficiency of the EMBR. By increasing the membrane flux to 105.6 L·m^−2^·h^−1^, the catalytic efficiency of the EMBR attained its peak value. However, a further increase in membrane flux decreased the activity of the EMBR, on account of the lessened reaction time between the substrate and immobilized enzyme. Therefore, sufficient reaction time and a proper flow rate to take away the catalytic product encourage the reaction to proceed positively are necessary conditions to ensure efficient catalytic performance of the EMBR.

The essence of the enzyme catalysis lies in the combination of enzyme and substrate, which accordingly reduces the activation energy of the reactants and increases the rate of reaction. Therefore, the reaction rate is largely dependent on the rate at which the enzyme substrate complex is formed. When the amount of immobilized enzyme is constant, the amount of complex formed per unit time is decided by the substrate concentration. As a consequence, the activity of the EMBR increased when the concentration of substrate increased and plateaued after 0.2 mol·L^−1^, as shown in Figure 9b.

Figure 9c shows that the activities of the free and immobilized enzyme both increased and then decreased with an increase in temperature, although the latter (43 °C) was more thermostable than the former (30 °C), which was consistent with the results as reported. After immobilization, the conformation of the enzyme is more difficult to change; therefore, a higher temperature is needed. Furthermore, mass transfer would increase correspondingly at the higher temperature.

### 2.8. Reusability of the EMBR

**Figure 10:**
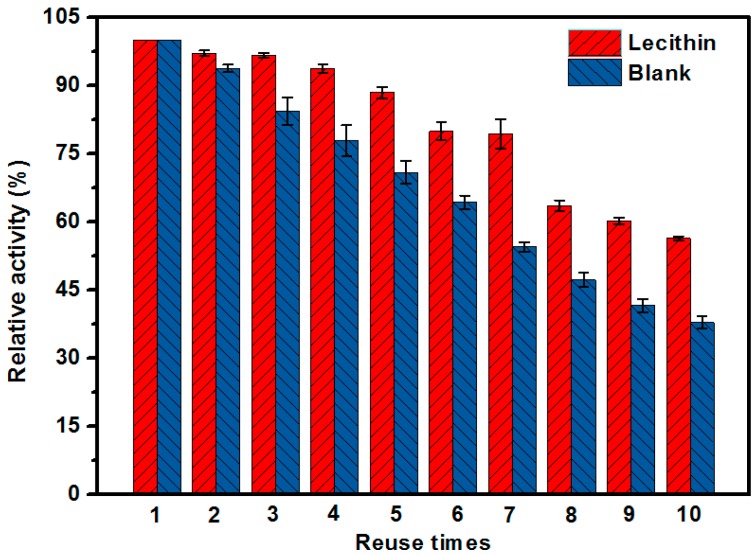
Reusability of the EMBR. (The two EMBRs were prepared under optimal conditions according to the above data. Test condition: 105.6 L·m^−2^·h^−1^ of membrane flux, 0.2 M substrate solution, T = 25 °C).

One of the most important advantages of enzyme immobilization is reusability, which is critical for practical large-scale applications. With constructions of the EMBR, the enzyme could be recovered and recycled easily. To study the reusability of the EMBR, the activity of the bioreactor was set at 100% in the first round, and the hydrolysis conversions were compared in subsequent cycles. As indicated in Figure 10, the activity of the EMBR with repeated use did not decrease significantly; the remaining activity was about 88% of the first use after five cycles, whereas the residual activity of the unmodified EMBR decreased to 70%, which indicated that the lecithin-modified EMBR had good durability.

The activity of two EMBRs decreased after several cycles because of the attenuate enzyme activity and the loss of enzymes from membrane might happen. Moreover, the thermal stability of the lecithin modified EMBR was better than the blank EMBR, in which the former relative activity was about 15% higher than the latter after 80 min reaction under the same condition (Supplementary Materials Figure S3).

## 3. Materials and Methods

### 3.1. Materials

Lecithin was purchased from Sinopharm Chemical Reagent Co. Ltd. (Shanghai, China). *Candida rugosa* lipase (CRL) powder (1150 units per mg solid), Bradford reagent, bovine serum albumin (BSA; molecular mass: 67 kDa), and glycerol triacetate were obtained from Sigma Aldrich Chemical Co. (St. Louis, MO, USA) and used as received. All other chemicals were of analytical grade and used without further purification. A hollow-fiber PSF microfiltration membrane with an average pore size of 0.236 μm was prepared with the common phase-inversion method [27]. The hollow-fiber membrane comprised perfect gradient pores, the size of which gradually decreased from the inner to the outer surface. It was then rinsed with deionized water and phosphate buffer solution (PBS; 0.05 M, pH 7.0) three times before use. The water permeability was subsequently measured at 0.02 MPa for 3 min.

### 3.2. The Determination of Optimal Surfactant to Enhance the Activity of Lipase

Surfactant solutions were prepared by dissolving an appropriate amount of surfactant solid (lecithin, betine, SDS, CTAB) in PBS (0.05 M, pH 7.0) with high-speed stirring until no solid particles were observed; the solution was then filtered through a syringe filter (0.22 μm) before use to obtain a completely dissolved surfactant solution. An aqueous solution (in PBS) of lipase was centrifuged at 4000 rpm for 8 min to remove insoluble impurities. Add 1.0 mL surfactant solution in the lipase solution, respectively. Then the mixed solution was poured into the glycerol triacetate. Acid would be produced through the catalysis of glycerol triacetate by lipase. The activity of the free enzyme was measured by automatic potentiometric titrator. The mixed solution was titrated with 0.1 M NaOH solution during the reaction to neutralize the produced acid to keep solution pH constant. According to the consumed amount of NaOH, the amount of produced acid could be obtained and thus the enzyme activity could be determined.

### 3.3. Preparation of an EMBR with Lecithin Biomimetic Interfaces

Lecithin solution was prepared according to Section 3.2. The prepared hollow-fiber PSF membrane module was placed in a dead-end filtration apparatus, and the lecithin solution was filtered through with continuous stirring under a flux of 102.6 L·m^−2^·h^−1^, by which means a biological affinity layer was formed on the surface of the skeleton of the PSF membrane.

**Figure 11:**
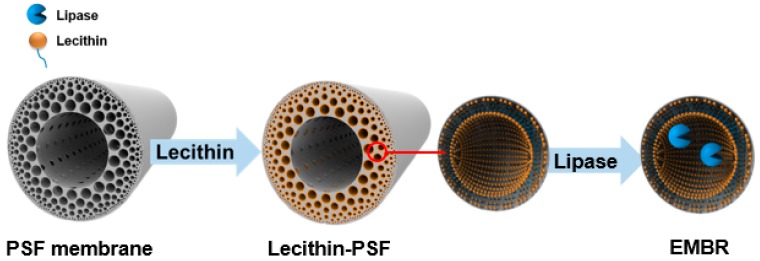
Schematic representation of lecithin modification and enzyme immobilization on the membrane.

An aqueous enzyme solution (in PBS) of lipase was centrifuged at 4000 rpm for 8 min to remove insoluble impurities. To immobilize the enzyme, the supernatant of the lipase solution was filtered through the lecithin-modified membrane under the same membrane flux for 2 h (Figure 11, Supplementary Materials Figure S2). Afterwards, the membrane containing immobilized enzymes was washed for 30 min with 40 mL of PBS under a higher flux of 197.7 L·m^−2^·h^−1^ to remove the weakly-adsorbed enzymes and the residual lecithin solution. The washings, together with the reaction solution, were collected for determination of the enzyme loading. The concentration of protein in the solution was determined by Bradford’s method [32]. BSA was used as a standard to construct a calibration curve. The enzyme loadings, which was defined as the amount of enzyme (mg) per gram of hollow-fiber membrane, was calculated from the protein mass balance among the initial and final lipase solutions and the washings. Each value was the mean of at least three parallel experiments, and the standard deviation was within approximately 5%.

A field-emission scanning electron microscope (FE-SEM, Hitachi, S4800, Tokyo, Japan) and liquid–liquid displacement porometer (LLP-1200A, Porous Materials Inc., Ithaca, NY, USA) were used to observe the morphology of a cross-section of the membrane surface, and the change of pore size before and after enzyme immobilization, respectively. EDX spectroscopy (Hitachi S4800) and fluorescence microscopy (Nikon, Ti-U, Tokyo, Japan) were used to characterize the existence of the immobilized enzymes.

### 3.4. Activity Assay for the EMBR

The activity of the EMBR was determined by dead-end filtration. In order to perform a continuous enzymatic hydrolysis process, 100 mL of glycerol triacetate aqueous solution were circulated through the inner-surface side of the membrane module by a peristaltic pump. To keep the reaction temperature constant and allow rapid diffusion of the produced acid, a water bath at a set temperature and magnetic stirring were provided. In the meantime, the substrate solution was titrated with 0.1 M NaOH solution by using an automatic titrator to keep the pH value constant during the whole process. The volume of consumed NaOH solution was recorded periodically and used to evaluate the reaction rate. The apparent enzymatic membrane activity corresponded to the release of 1 mmol acetic acid per min per gram of the membrane under the assay conditions. The apparent enzyme activity was defined as the amount of acetic acid produced per min per milligram of lipase under the assay condition. Each data point was the average of at least three parallel experiments, and the standard deviation was within 5%. Blank experiments were performed without immobilized lipase on the membrane at pH 7.0 and temperatures from 15 to 55 °C to allow the effect of self-hydrolysis to be deducted.

### 3.5. Reusability of the EMBR

To evaluate the reusability, the tested immobilized-lipase membranes were washed with PBS to remove the residual substrate and product after the hydrolysis reaction; this was followed by filtering of fresh substrate solution under the same experimental conditions. The hydrolysis reactions were conducted at 25 °C with 0.2 M glycerol triacetate solution as the substrate under the optimal membrane flux. The same procedure was repeated up to eight times. The relative catalytic activities of the immobilized-lipase were normalized to the highest activity.

## 4. Conclusions

On the basis of the cell fluid mosaic model, a facile method integrating a biomimetic interface and immobilization was developed to construct an EMBR with enhanced performance. The immobilized-lipase membrane bioreactor was prepared by immobilizing lipase on a lecithin-modified PSF hollow-fiber membrane with perfect radial gradient pores through facial dead-end filtration. The performance of the EMBR was studied by using hydrolysis of glycerol triacetate as a model reaction and operational variables such as membrane flux, substrate concentration and temperature were investigated to determine the optimal experimental conditions. As a result, the prepared EMBR showed excellent catalytic performance, with 42% improved enzymatic activity and 78% improved catalytic efficiency relative to a blank membrane. Furthermore, the remaining activity was about 88% of the first use after five cycles, which indicated that the EMBR had good durability. In conclusion, immobilization of enzymes on PSF membranes with specific microstructures and a biomimetic interface modified by lecithin can achieve increased enzyme activity and membrane catalytic efficiency. Therefore, this EMBR has a great potential in practical application, such as catalyzing a series of diverse reactions, including hydrolysis, alcoholysis, aminolysis and transesterification with various organic substrates. Meanwhile, such construction system of EMBR is also applicable for other kinds of enzymes.

## Figures and Tables

**Table 1:** Analysis of elemental content of the lecithin-modified PSF membrane. (Mean value of three times measurements, the standard deviation was within 5%).

C_Lecithin_ (mg·mL^−1^) Elements	C	N	O	S
0.00	82.90%	0.00%	14.29%	2.82%
0.25	77.49%	4.19%	14.77%	3.55%
0.50	75.78%	4.26%	15.76%	4.20%
0.75	77.05%	4.86%	14.39%	3.70%

## Supplementary Materials

The supplementary materials are available online.
