# Supplementary material for: Immobilization of Enzymes on a Phospholipid Bionically Modified Polysulfone Gradient-Pore Membrane for the Enhanced Performance of Enzymatic Membrane Bioreactors
Source: Molecules. 2018 Jan 11;23(1):144. doi: 10.3390/molecules23010144 (PMC6017099; doi:10.3390/molecules23010144)
Supplement: Supplementary file 1 [file molecules-23-00144-s001.pdf]

Support information:

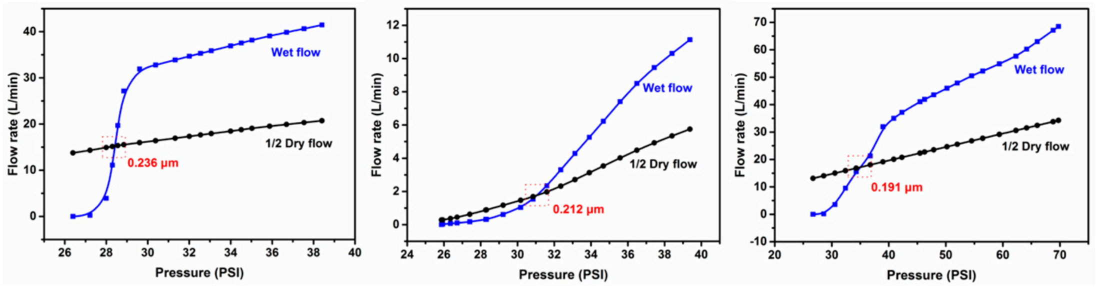

Figure S1. Sketch of the pressure-flow rate curves measured by LLP. (a) Blank RGM-PSF membrane; (b) Lecithin-modified RGM-PSF membrane; (c) Lipase immobilized RGM-PSF membrane;

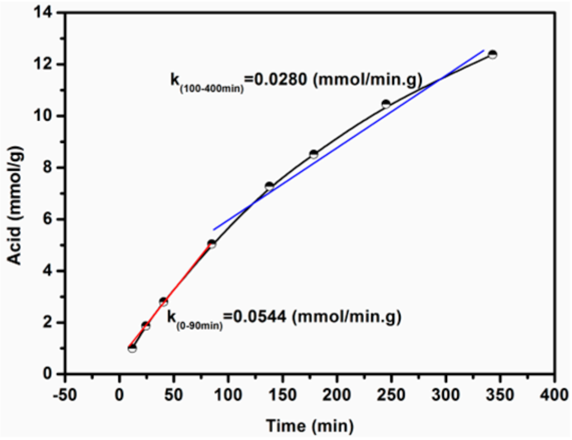

Figure S2. Time course of TA hydrolysis in the bioreactor.

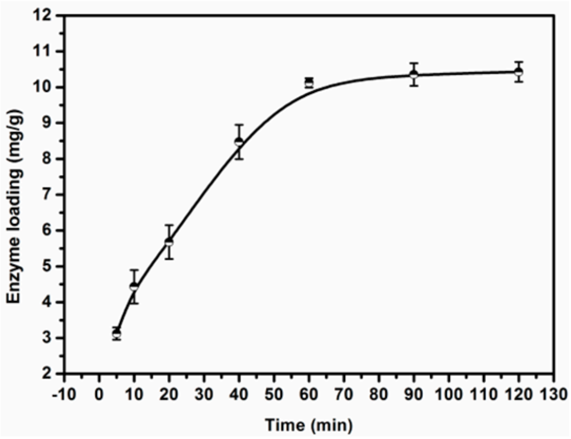

Figure S3. Effect of time on the enzyme loading.

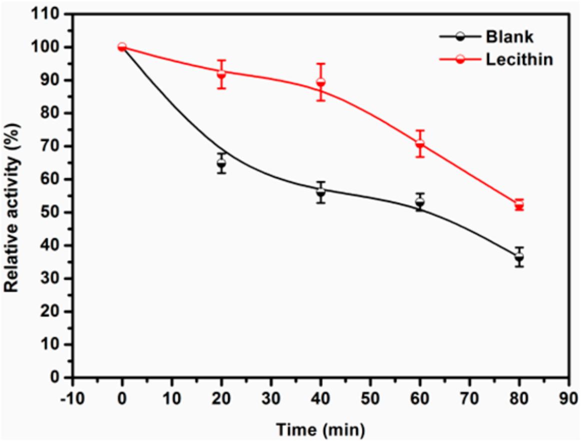

Figure S4. Thermal stability of the lecithin-modified EMBR and blank EMBR.

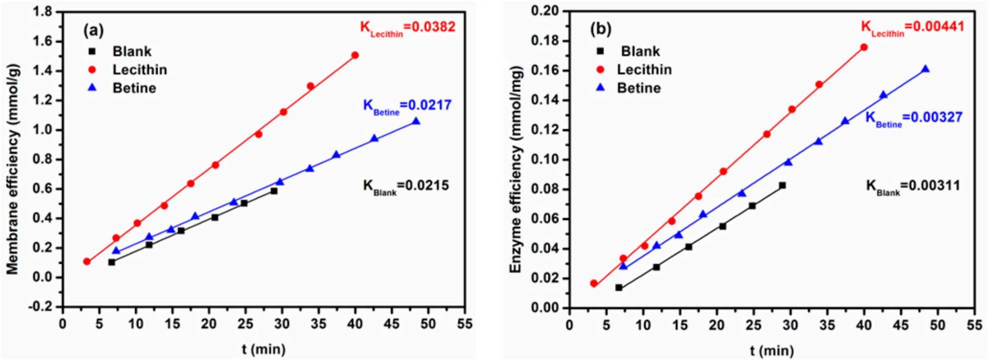

Figure S5. The comparision of the activity of membrane and enzyme between different surfactants modified EMBRs.
